# Functional integration of Cas9 gene into the genome of rhesus monkey: possibility of a new biomedical model?

**DOI:** 10.1093/lifemedi/lnad002

**Published:** 2023-01-18

**Authors:** Yi Wang, Junmo Wu, Xuan Wang, Yu Kang, Chu Chu, Yuqiang Zeng, Zhenzhen Chen, Ziyi Zhao, Xinglong Chen, Baohong Tian, Yuyu Niu

**Affiliations:** State Key Laboratory of Primate Biomedical Research, Institute of Primate Translational Medicine, Kunming University of Science and Technology, Kunming 650500, China; Yunnan Key Laboratory of Primate Biomedical Research, Kunming 650500, China; State Key Laboratory of Primate Biomedical Research, Institute of Primate Translational Medicine, Kunming University of Science and Technology, Kunming 650500, China; Yunnan Key Laboratory of Primate Biomedical Research, Kunming 650500, China; State Key Laboratory of Primate Biomedical Research, Institute of Primate Translational Medicine, Kunming University of Science and Technology, Kunming 650500, China; Yunnan Key Laboratory of Primate Biomedical Research, Kunming 650500, China; State Key Laboratory of Primate Biomedical Research, Institute of Primate Translational Medicine, Kunming University of Science and Technology, Kunming 650500, China; Yunnan Key Laboratory of Primate Biomedical Research, Kunming 650500, China; State Key Laboratory of Primate Biomedical Research, Institute of Primate Translational Medicine, Kunming University of Science and Technology, Kunming 650500, China; Yunnan Key Laboratory of Primate Biomedical Research, Kunming 650500, China; State Key Laboratory of Primate Biomedical Research, Institute of Primate Translational Medicine, Kunming University of Science and Technology, Kunming 650500, China; Yunnan Key Laboratory of Primate Biomedical Research, Kunming 650500, China; State Key Laboratory of Primate Biomedical Research, Institute of Primate Translational Medicine, Kunming University of Science and Technology, Kunming 650500, China; Yunnan Key Laboratory of Primate Biomedical Research, Kunming 650500, China; State Key Laboratory of Primate Biomedical Research, Institute of Primate Translational Medicine, Kunming University of Science and Technology, Kunming 650500, China; State Key Laboratory of Primate Biomedical Research, Institute of Primate Translational Medicine, Kunming University of Science and Technology, Kunming 650500, China; Yunnan Key Laboratory of Primate Biomedical Research, Kunming 650500, China; State Key Laboratory of Primate Biomedical Research, Institute of Primate Translational Medicine, Kunming University of Science and Technology, Kunming 650500, China; Yunnan Key Laboratory of Primate Biomedical Research, Kunming 650500, China; State Key Laboratory of Primate Biomedical Research, Institute of Primate Translational Medicine, Kunming University of Science and Technology, Kunming 650500, China; Yunnan Key Laboratory of Primate Biomedical Research, Kunming 650500, China; Faculty of Life Science and Technology, Kunming University of Science and Technology, Kunming 650500, China

Dear Editor,

In this study, we described the generation of a Cas9-cop GFP-expressing cynomolgus monkey fibroblast cell line via a human immunodeficiency type 1(HIV-1)-based vector. We further demonstrated convenient and efficient utilization of the cell line in targeted editing of specific genes solely using delivery of single-guide RNA (sgRNA). In order to facilitate broader applications of CRISPR-Cas9, we also attempted to generate a Cas9 transgene rhesus monkey to overcome the delivery challenges associated with Cas9 to provide an attractive model for studying biological processes and diseases.

To introduce the Cas9 gene into the female cynomolgus monkey fibroblast, we used a HIV-1-based vector as a transgene. The vector consists of a FLAG-tagged *Streptococcus pyogenes* Cas9 driven by the CAG promoter and a green fluorescent protein (cop GFP) driven by the EF1-a promoter to facilitate visualization of Cas9-expressing cells (Fig. 1A). The procedure for lentivirus packaging is described in the Methods. After the preliminary verification of the lentivirus, we infected the cynomolgus monkey fibroblast cell line with the lentivirus. When the fibroblast cell density reached 60%–70%, we added lentivirus to the medium. We can detect fibroblast fluorescence at 48 h post transduction (Fig. 1B). The cells have a low-transduction efficiency, where only 8.17% positive cells could be harvested. It was therefore necessary to purify the infected cells. Because the lentivirus also expressed cop GFP, the cop GFP-positive cells were sorted and screened by flow cytometry. After three rounds of flow cytometry sorting, we obtained a fibroblast cell line with a cop GFP-positive rate of ~70% (Fig. 1B and 1C), which was used in the subsequent experiments.

**Figure 1.**
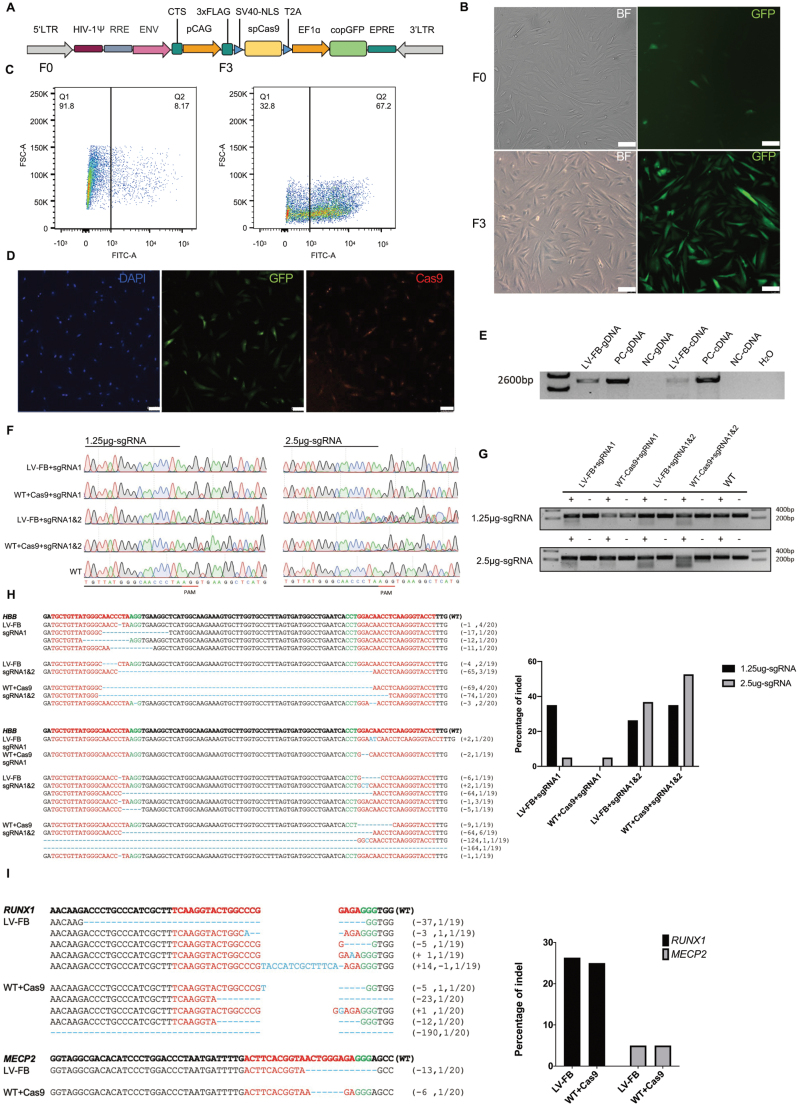
Generation and identification of Cas9 expressing cell line. (A) Schematic of the Cas9-cop GFP Lentivirus vector. (B) Fluorescence and bright field images of fibroblasts 48 h after lentivirus infection (F0) and three times after flow cytometry sorting (F3). (Scale bar: 100 μM.) (C) Flow cytometry analysis of cop GFP expression in fibroblasts 48 h after lentivirus infection (F0) and three times after flow cytometry sorting (F3). (D) Immunofluorescence staining of LV-FB, showing the Cas9-P2A-EGFP expression. The fibroblast from a normal monkey was performed as control. (Scale bar: 100 μM.) (E) PCR analysis of genomic DNA and cDNA showed the Cas9 insertion and expression of infected fibroblast. Using a commercial Cas9-expressing HEK293 cell as positive control, a fibroblast from a normal monkey as negative control. (F) Sanger sequencing results of sgRNA targeting sites, showing the cleavage effect of infected fibroblast is functional. (G) T7 Exonuclease cleavage assay was performed to test the cleavage effect of LV-FB, representing the functional Cas9 endonuclease existing in the lentivirus-infected cells. Using the fibroblast from a normal monkey adding extra Cas9 protein was used as control. (H) Sequences of modified HBB loci detected in LV-FB, and the fibroblast from a normal monkey adding extra Cas9 protein was used as control. At least 19 TA clones of the PCR products were analyzed by Sanger sequencing. The PAM sequences are underlined and highlighted in green; the sgRNA targeting sequences in red; the mutations in blue; deletions (−), insertions (+). N/N indicates positive colonies out of total sequenced. Graph showing the percentage of indel in two-cell line. (I) Sequences of modified RUNX1 and MECP2 loci detected in LV-FB, and the fibroblast from a normal monkey adding extra Cas9 protein was used as control. At least 19 TA clones of the PCR products were analyzed by Sanger sequencing. Graph showing the percentage of indel in two-cell line.

The co-expression of Cas9 and cop GFP were also proved by immunofluorescence analysis (Fig. 1D). To evaluate the expression of the Cas9, we extracted the DNA and RNA from the cells and confirmed by PCR (Fig. 1E). To confirm the cell lines are capable of editing, previously described sgRNA targeting the genes *HBB* [1]*, RUNX1,* or *MeCP2* was delivered into the cells via the nucleofection (Fig. 1F). As a control, CRISPR-Cas9 RNP was transiently introduced into wild type cynomolgus monkey fibroblasts. For *HBB*, we tested the editing efficiency of one sgRNA and two sgRNAs in the same cell line, and we also detected the effect of 1.25 and 2.5 μg sgRNA concentrations on editing efficiency respectively. Through the setting 1–2 sgRNAs for the wild type + CRISPR-Cas9 RNP and the Cas9 express cell line, the editing effect of lentivirus-infected fibroblast (LV-FB) and control cell in different conditions can be more clearly compared. sgRNA or RNP were delivered into the cells by a Lonza 4D nuclear transfection instrument, and the cells were collected 48 h after transfection for genome extraction. Sanger sequencing of the targeted site (Fig. 1F) showed that single sgRNA transfection in both the experimental group and the control group of transient Cas9 addition show less efficiency as compared to two sgRNA, where editing was then achieved in both control and experimental groups. The T7 Exonuclease cleavage assay (T7 Endo I, T7EI) also showed editing effect (Fig. 1G), and the editing effect produced by the virus infected Cas9 cell lines and the control cell lines was similar under the same editing condition. After increasing the concentration of sgRNA, part of the editing efficiency of both LV-FB and control cells were improved. Editing efficiency was further assessed by TA cloning (Fig. 1H and 1I), which prompted us to extend the work to monkeys and attempt to generate rhesus monkey-specific systems stably expressing Cas9 which enable Cas9/sgRNA-assisted homology-directed gene editing.

To broadly enable the application of the Cas9 system for gene editing *in vivo*, we tried to establish a Cas9-expressing rhesus monkey. We decided to use the lentivirus above-mentioned in a previous described approach [2] of infecting early-cleavage-stage embryos (Fig. 2A). We injected 50–100 pL virus suspension into the perivitelline space, and a total of 52 *in vitro*-fertilized embryos were used, consisting of 19 at one- or two-cell stage, 9 at four- to eight-cell stage, and 24 at morula- to blastula-stage (Fig. 2B). After 3–5 days culturing, we determined the expression of Cas9 and EGFP in the injected embryos by PCR. The EGFP-positive embryos were transplanted across 16 surrogate mothers, with 4 of them achieving successful implantation. One recipient miscarried on day 93, and one prematurely delivered. We ultimately obtained three transgenic baby monkeys from embryos infected at morula- to blastula-stage (Fig. 2C).

**Figure 2.**
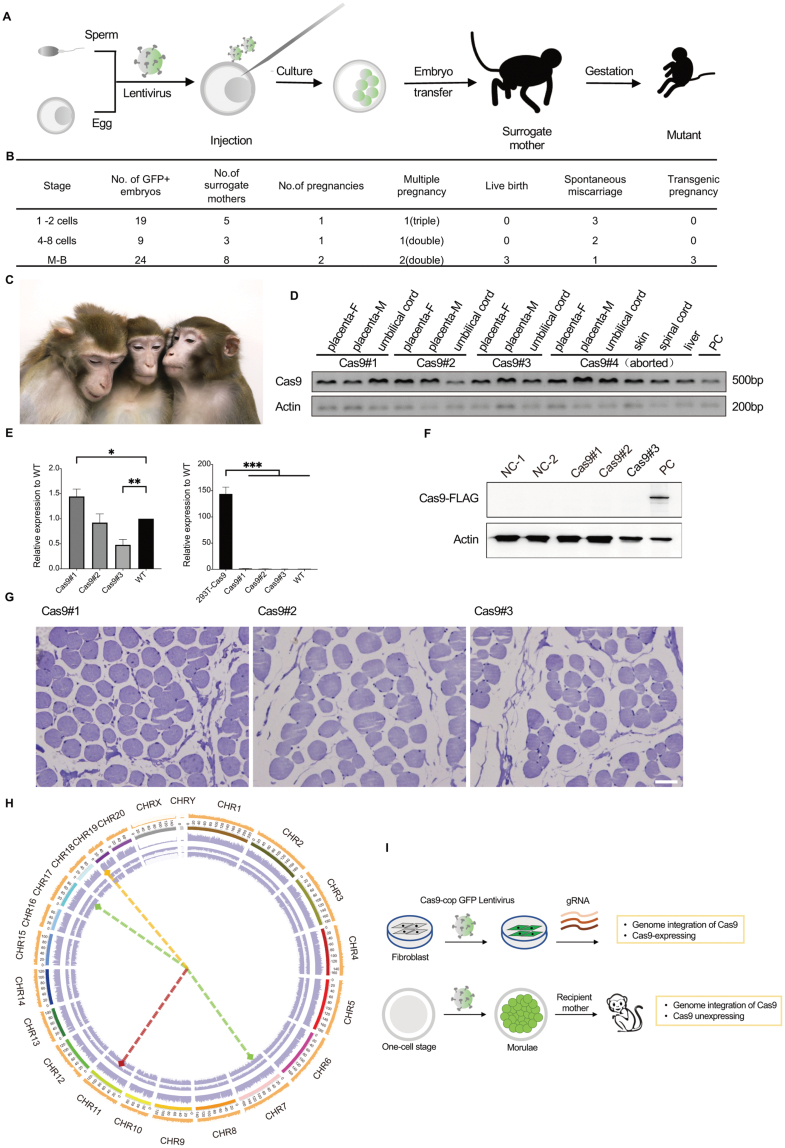
Generation and identification of lentivirus-mediated transgene monkey. (A) The schematic showed the process of generating Cas9 monkeys. (B) Pregnancy, live births, and transgenic outcomes. (C) Photographs of transgene monkeys when they were 5-years old. (D) PCR analysis of genomic DNA showing the presence of the Cas9 transgene in 3 (or 6) different organs of the 3 live fetus and an aborted one. Using a commercial Cas9-expressing HEK293 cell as positive control. (E) Quantitative analysis of Cas9 mRNA expression in the Cas9 monkey fibroblasts and commercial Cas9-expressing HEK293 cell by RT-qPCR. The data from the Cas9 monkeys were normalized to the corresponding data obtained from the WT monkeys. Data shown as mean ± SD, *n* = 3 wells per condition, *P < 0.05, **P < 0.01, ***P < 0.001 (*t*-test). (F) Western blots showed the not expression of Cas9 in the fibroblasts. (G) Immunohistochemistry of the muscle showed no Cas9 expression in the three monkeys. The muscle from a normal monkey was performed as control. (Scale bar: 50 μM.) (H) Circos maps showed the integration site of each monkey and the amount of genomic variation, the purple stripe represents Cas9 #1, Cas9 #2, and Cas9 #3 from the outside to the inside, the yellow stripe represents wild type. (I) Graphical summary.

The integration and expression of Cas9 within the transgenic fetus and three baby monkeys were examined in different tissues. In addition to testing the placenta and umbilical cord, aborted fetus are also tested for the integration of the Cas9 gene in the skin, spinal cord, and liver. (Figs. 2D and S1A–C). All tissues tested had Cas9 integration and expression of Cas9 mRNA, as confirmed by PCR and RT-PCR, and when tested the baby monkeys skin fibroblast, it showed the same result. These results indicated that the Cas9 successfully inserted into the genome of those monkeys and also transcribed into mRNA. However, in comparison to commercial Cas9-expressing HEK293 cells, the relative protein expression level of the Cas9 in the monkeys is almost negligible (Fig. 2E). When three rhesus monkeys were 3 years old, we further assessed protein in fibroblasts from the monkeys by Western blot (Fig. 2F) and did not observe expression of the Cas9 protein. This result was also verified by immunohistochemistry of muscle from the animals (Fig. 2G). We also used electroporation to deliver HBB sgRNA to test the function of endogenic Cas9 nuclease and failed to introduce any mutation (data not shown).

In order to explore the reason why Cas9 protein was not produced in these monkeys, we generated 116 Gbp (~40X) whole-genome shotgun (WGS) data from the blood of the animals and blasted the linear DNA [the sequence between the two tandem copies of the long terminal repeat (LTR) sequence] within the genome to search for the integration site of Cas9-P2A-EGFP. The results showed that the three transgenic monkeys had only 1 or 2 complete integration sites, all within intergenic regions (Figs. 2H and S1D). The causes of low-integration number of the complete desired sequence, which is well below expectation, may on account of two factors: the immune restriction of the lentivirus and the large size of Cas9.

Using lentivirus-based delivery of Cas9 as an approach, we have integrated Cas9 into fibroblasts and individual rhesus monkeys. The fibroblast cell line express functional Cas9 protein able to edit loci in the presence of gRNAs, whereas the rhesus monkeys only express Cas9 mRNA and do not produce Cas9 protein.

The entry of foreign DNA into many mammalian cell types triggers the innate immune system, a complex set of responses directed at preventing infection by pathogens. To date, retroviral restriction is still considered a complex phenomenon that may limit gene therapy applications. To protect their genomic integrity, animals control retroviral infections by establishing heritable epigenetic silencing of the integrated provirus in early embryonic development [3]. Some remarkable progress have emphasized that DNA methylation [4], histone modification [5], provirus copy number, and integration site preferences [6] are the key mechanisms for lentivirus vector silencing. However, in our study, three transgene monkeys still being transcribed and RNA produced, we hypothesized that the mRNA could not be translated into Cas9 protein due to dsRNA-mediated silencing mechanism [5]. Several β-globin gene therapy papers have demonstrated that wild type and SIN lentivirus expression can be variegated or inconsistent when the provirus copy number is <3 [7]. It was also shown that the HIV-1 LTR and *gag* sequences can silence linked LCR β-globin transgenes after DNA microinjection into fertilized mouse eggs [8]. Based on the above analysis, we speculated that the low-integration number and lentiviral suppression resulted in non-expression of the Cas9 transgene.

In our work towards generating a Cas9 expressing rhesus monkey model, the WGS data analysis and the integration site information provides a reference for generating primate model by lentivirus injection into early-cleavage-stage embryos. It also highlights that when using lentivirus in cells such as embryonic or stem cells with differentiation potential, it is necessary to consider the size and characteristics of the integrated sequence, as well as the immunogenicity of stem cells to retroviruses. Meanwhile, choose a safe harbor for gene knock in should be considered when generating animal models [9]. These factors will influence the efficiency of lentiviral integration and the success of target gene expression.

In addition, some studies have shown that there are preexisting humoral and cell-mediated adaptive immune responses to Cas9 in humans, which is likely to prevent the safe and effective use of CRISPR/Cas9 system in the treatment of diseases, and may even cause significant toxicity to patients [10]. This may also be an explanation for lack of Cas9 protein expression we observed in the Cas9 transgenic monkeys.

## Research limitations

Our study described the generation of a Cas9-cop GFP-expressing cynomolgus monkey fibroblast cell line. We also attempted to generate a Cas9 transgene rhesus monkey to overcome the delivery challenges associated with Cas9 to provide an attractive model for studying biological processes and diseases. Stable integration of Cas9 was observed in the monkey genome, however, Cas9 protein expression was not detected.

## Supplementary Material

lnad002_suppl_Supplementary_Figure_S1

lnad002_suppl_Supplementary_Material
