# Supplementary figures and images for: Functional integration of Cas9 gene into the genome of rhesus monkey: possibility of a new biomedical model?
Source: Life Med. 2023 Jan 18;2(1):lnad002. doi: 10.1093/lifemedi/lnad002 (PMC11749692; doi:10.1093/lifemedi/lnad002)

Supplement Figure 1

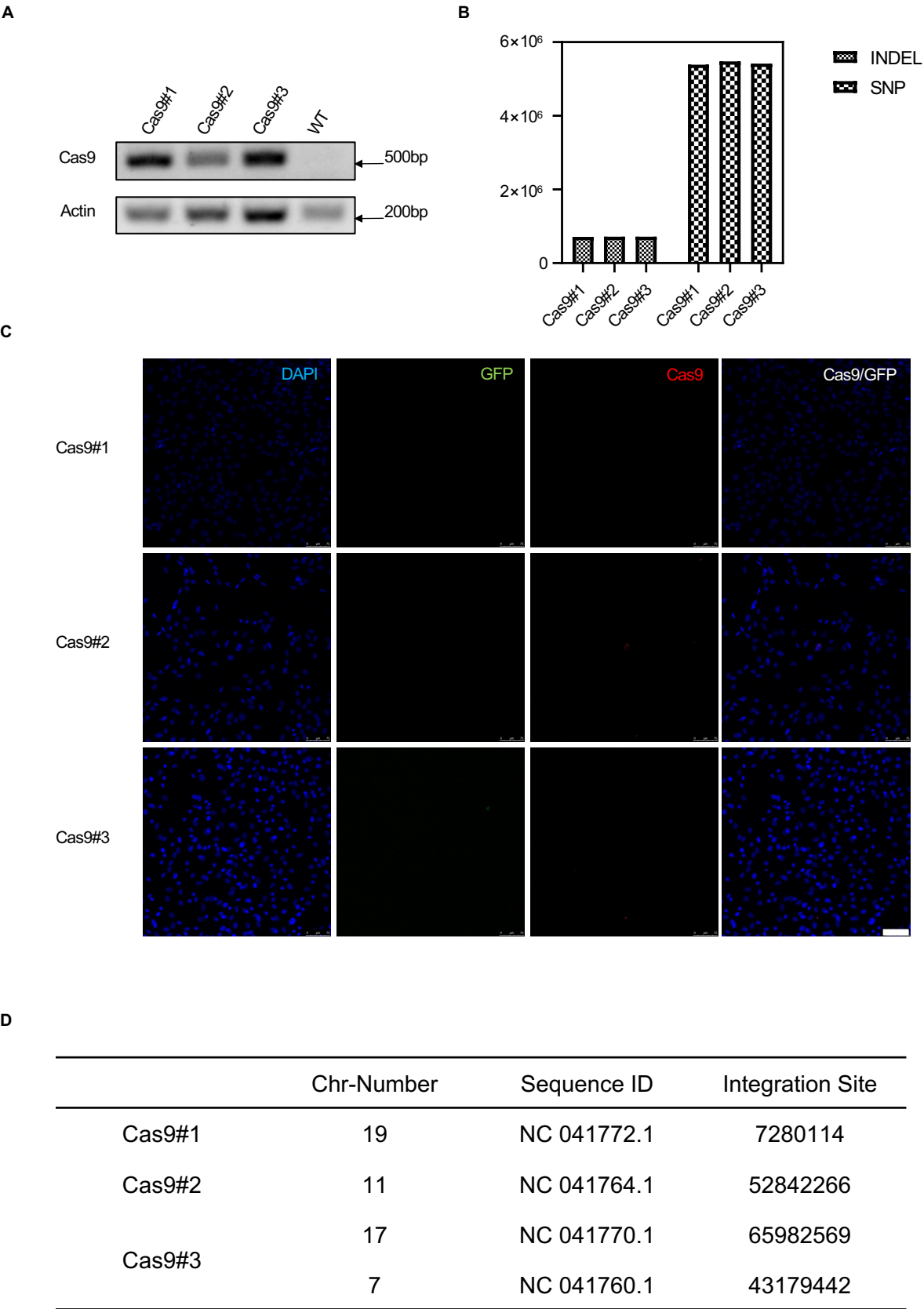

Supplement: lnad002_suppl_Supplementary_Figure_S1 [file lnad002_suppl_Supplementary_Figure_S1.pdf]
