# Supplementary material for: Functional integration of Cas9 gene into the genome of rhesus monkey: possibility of a new biomedical model?
Source: Life Med. 2023 Jan 18;2(1):lnad002. doi: 10.1093/lifemedi/lnad002 (PMC11749692; doi:10.1093/lifemedi/lnad002)
Supplement: lnad002_suppl_Supplementary_Material [file lnad002_suppl_Supplementary_Material.docx]

**Supplementary Information**

**Functional integration of Cas9 gene into the genome of rhesus monkey: possibility of a new biomedical model?**

**Materials and methods**

**Animals**

Healthy Chinese rhesus monkey (*Macaca mulatta*) (7-17 year-old) were used in this study. All animals were housed at the State Key Laboratory of Primate Biomedical Research (LPBR). All animal procedures were performed following the association for Assessment and Accreditation of Laboratory Animal Care International (AAALAC) for the ethical treatment of primates.

**Collection of rhesus monkey oocytes and in vitro fertilization**

Assisted reproductive techniques in rhesus monkey have been described elsewhere (Niu et al. 2010). In brief, we collect oocyte from female rhesus monkey age 5-8 years, and performed successively intramuscular injection with recombinant human FSH (rhFSH) (Gonal FTM Laboratories), human chorionic gonadotropin (hCG) (Lizhu Groups) to follicular stimulation 32-35 h after rhCG administration, we collected the oocytes by laparoscopic follicular aspiration and then put the follicular contents in Hepes buffered Tyrode’s albumin lactate pyruvate (TALP) medium containing 0.3% BSA at 37°C. Using the hyaluronidase (0.5 mg/mL) to remove the cumulus cells and classified the oocytes with nuclear maturity into three classes. Oocytes that were mature (MII) at collection were used to fertilization with hyperactivated spermatozoa which final concentration of 2 × 10^5^ /mL. Fertilization was confirmed by the presence of the second polar body and two pronuclei. Embryos at the 1-2 cell stage, 4-8 cell stage and morula- to blastula-stage were respectively selected for embryo transfer. Surrogate females exhibiting normal menstrual cycles were identified based on their steroid hormone profiles and observation of menses. Embryo transfer into the oviduct was conducted by the laparoscopic approach.

**Cell culture**

HEK293 cells were grown in Dulbecco’s modified Eagle’s medium (DMEM) (Thermo Fisher Scientific, USA) supplemented with 10% fetal bovine serum (Biological Industries，Israel), 1% Pen-Strep Solution (Thermo Fisher Scientific, USA) and grown at 37°C in 5% CO_2_. Macaca fibroblast cells were grown in Dulbecco’s modified Eagle’s medium (DMEM) supplemented with 10% fetal bovine serum, 1% Minimum Essential Medium (MEM) non-essential amino acids solution (Thermo Fisher Scientific, USA), 1% Pen-Strep Solution and grown at 37°C in 5% CO_2_. HEK293 cells and monkey fibroblast cells were digested by 0.05% trypsin-EDTA (Thermo Fisher Scientific, USA) and collected by centrifugation at 150 *g* for 5 minutes.

We are pleased to share fibroblast cell lines expressing Cas9 protein who wants to get this cell can contact corresponding authors.

**Viral vector design and lentivirus production**

The lentivirus backbone vector was obtained from SBI (CD511B-1) and the PX458 plasmid (Addgene, #104041) was used as the donor vector for the Cas9 sequence. After annealing, the Cas9 segments were inserted into the SpeⅠand SalⅠ site of the backbone vector. The lentivirus was constructed in HEK293 (American Tissue Culture Collection) cells according to the method described elsewhere. Briefly, HEK293 cells were seeded in a dish 15 cm in diameter 24 h before transfection and ensure the cell fusion reached 60% the next day. Cells were transfected by PEI method with a mixture of DNAs containing 30 μg Vector DNA, 20 μg PMDL, 11 μg REV, 8 μg VSVG. Added opti-MEM (Thermo Fisher Scientific, USA) to 2 mL and mixed, incubated for 5 min.In addition, a centrifuge tube was used to add PEI solution with triple plasmid volume. The following day, cells were fed again with fresh medium and further cultured for 48-72 h. The supernatant then was clarified by centrifugation (1,000× *g*, 5 min), passed through a cellulose acetate filter (pore size, 0.45 μm), and concentrated by ultracentrifugation (40,000× *g*, 1 h). The viral pellet was resuspended in DMEM, frozen.

**PCR and RT-PCR**

The genomic DNA was extracted from total blood and tissues by Wizard Genomic DNA Purification Kit (Promega, #A1125) and RNA was extracted by Invitrogen TRIzol. To detect the Cas9 gene, forward primer (5’-GATACACCAGACGGAAGAACC-3’) and reverse primer (5’-CGCTAATCTGGACAAAGTGC-3’) was used to performed Sanger sequencing after PCR, forward primer (5’-GACCCTAAGAAGTACGGCGG-3’) and reverse primer (5’-TTGTAGCCCTTGGCTTCCAG-3’) was used to performed RT-PCR.

**Western blot**

Total proteins were extracted from fibroblast and their concentration was determined using the Enhanced BCA Protein Assay Kit (Beyotime). Equal amounts of protein extracted with loading dye were boiled before loading into 4-15% gradient polyacrylamide gels (Bio-Rad). Proteins were transferred onto a PVDF membrane (Bio-Rad) after electrophoresis. And then blocking in 5% skimmed milk for 2 h, incubating with the Cas9 primary antibodies (abcam, 1:1,000 dilution) and β-actin (proteintech, 1:5,000 dilution), followed by secondary (H+L) HRP conjugate antibodies (Sigma-Aldrich) for detecting proteins with a Chemiluminescent HRP substrate (Millipore).

**Immunofluorescence staining**

The cells were fixed with 4% paraformaldehyde for 10 minutes, permeated with 0.1% Triton X-100 in PBS, and sealed with 3% BSA for 1 hour. We used Formalin-Fixed and Paraffin-Embedded (FFPE) for the tissue immunohistochemistry (IHC), antigen repair was performed after section. Subsequently, the cells/tissues were incubated with Cas9 primary antibodies (abcam) overnight at 4°C, washed with PBS three times, followed by incubation with secondary antibodies (goat anti-mouse, Invitrogen) for one hour and washed with PBS three times.

**Verification of Cas9 cleavage effects**

GeneArt Precision gRNA Synthesis Kit (Thermo Fisher Scientific, USA) was used to amplify SgRNA oligos and transcribe in vitro, then purified with the MEGAclear Kit (Thermo Fisher Scientific, USA) according to the manufacturer’s instructions. To contrast with Cas9-expressing fibroblast, TrueCut™ Cas9 Protein v2 (Thermo Fisher Scientific, USA) incubated with the sgRNA to form ribonucleoprotein complexes (RNP) were transfected into cells by electroporation using 4D-Nucleofector (Lonza, Swiss). T7 EndonucleaseⅠ(NEB) was used to test the cleavage effect.

**Whole genome shotgun analysis**

In order to identify the integration site of Cas9-P2A-EGFP and the potential genetic structural variation caused by lentivirus transfection, we performed high-depth (30×) sequencing on the three Cas9-lentivirus transfected macaques and one wild-type macaque. We used the following procedures to analysis: FastQC-check the sequencing quality of offline data, FastP-remove low-quality sequencing bases from the joint box, BWHA-mem-compare the offline quality-controlled data to the macaque genome (MMUL_10), SamTools-convert SAM files into BAM files, and Pindel-extracted variation files from BAM files. Circos-for the visualization of variation data.

**Data availability**

The raw sequence data reported in this paper have been deposited in the Genome Sequence Archive in BIG Data Center, Beijing Institute of Genomics (BIG), Chinese Academy of Sciences, under accession number CRA013108 that are publicly accessible at <https://bigd.big.ac.cn/gsa>.

**Figure S1. Generation and identification of lentivirus-mediated transgene monkey.**

(A) PCR analysis of cDNA showed the Cas9 expression of the three Cas9 monkeys. Using a fibroblast from a normal monkey as negative control. (B) Immunofluorescence staining of fibroblast Induced by the three monkeys, showing no Cas9 expression. (Scale bar: 75 μM.) (C) Somatic variation statistics of three monkeys compared to wild type, it shows no statistical difference. The fibroblast from a normal monkey was performed as control. (D) Detail information of the integration site of each monkey. Chr, Chromosome.
